# Computational Analyses and Challenges of Single-cell ATAC-seq

**DOI:** 10.1093/gpbjnl/qzaf115

**Published:** 2025-11-21

**Authors:** Chenfei Wang, Jiaojiao Zhou, Hong Zhang, Zihan Zhuang, Gali Bai, Ming Tang, Song Liu, Tao Liu

**Affiliations:** Key Laboratory of Spine and Spinal Cord Injury Repair and Regeneration of Ministry of Education, Department of Orthopedics, Tongji Hospital, School of Life Science and Technology, Tongji University, Shanghai 200092, China; Frontier Science Center for Stem Cells, School of Life Sciences and Technology, Tongji University, Shanghai 200092, China; Department of Biostatistics and Bioinformatics, Roswell Park Comprehensive Cancer Center, Buffalo, NY 14263, USA; Department of Biostatistics and Bioinformatics, Roswell Park Comprehensive Cancer Center, Buffalo, NY 14263, USA; Department of Computational Biology, College of Agriculture and Life Sciences, Cornell University, Ithaca, NY 14850, USA; Department of Data Science, Dana-Farber Cancer Institute, Boston, MA 02215, USA; Department of Data Science, Dana-Farber Cancer Institute, Boston, MA 02215, USA; Department of Biostatistics and Bioinformatics, Roswell Park Comprehensive Cancer Center, Buffalo, NY 14263, USA; Department of Biostatistics and Bioinformatics, Roswell Park Comprehensive Cancer Center, Buffalo, NY 14263, USA

**Keywords:** scATAC-seq, Epigenetic landscape, Gene regulation, Computational analysis, Multiomics integration

## Abstract

Single-cell Assay for Transposase-Accessible Chromatin using sequencing (scATAC-seq) has emerged as a powerful technique to study cell-specific epigenetic landscapes and to provide a multidimensional portrait of gene regulation. However, low genomic coverage per cell results in intrinsic data sparsity and missing-data issues, presenting unique methodological challenges. Consequently, numerous computational methods and techniques have been developed to address these challenges. This review provides a concise overview of published workflows for scATAC-seq analysis, covering preprocessing through downstream analysis including quality control, alignment, peak calling, dimensionality reduction, clustering, gene regulation score calculation, cell type annotation, and multiomics integration. Additionally, we survey key scATAC-seq databases that offer curated, accessible resources; discuss emerging deep-learning methods and Artificial Intelligence (AI) foundation models tailored to scATAC-seq data; and highlight recent advances in spatial ATAC-seq technologies and associated computational approaches. Our objective is to equip readers with a clear understanding of current scATAC-seq methodologies so they can select appropriate tools and construct customized workflows for exploring gene regulation and cellular diversity.

## Graphical abstract

**Figure:**
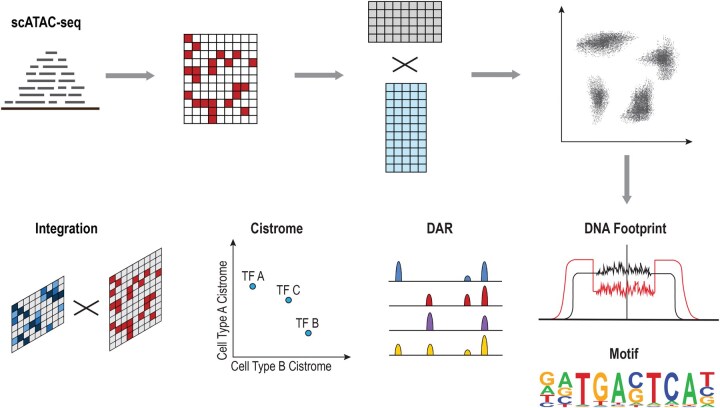

## Introduction

Studying chromatin accessibility is crucial as it reveals regulatory mechanisms controlling gene expression and cellular identity. Assay for Transposase-Accessible Chromatin using Sequencing (ATAC-seq), which profiles genome-wide chromatin accessibility by inserting sequencing adapters into open chromatin regions via a Tn5 transposase, has emerged as the most popular method due to its simplicity and sensitivity [1]. However, bulk ATAC-seq measures the averaged chromatin landscape of pooled cells, obscuring cell-to-cell variability. Recent advances in single-cell genomics have enabled the interrogation of cellular behavior at unprecedented resolution [2]. Its adaptation on ATAC-seq, known as single-cell ATAC-seq (scATAC-seq), was introduced in 2015, enabling the analysis of chromatin accessibility at an individual cell level [1,3]. A range of scATAC-seq platforms has since been developed, including nanowell-based (ICELL8), microfluidics-based (Fluidigm C1), droplet-based (10X Genomics Chromium), and combinatorial indexing-based approaches, each with distinct advantages and trade-offs discussed in a dedicated review [4].

However, the relatively small number of sequenced DNA fragments obtained from each cell, when compared to bulk ATAC-seq [5] can lead to intrinsic sparsity in scATAC-seq data, posing unique challenges for data analysis and interpretation [6]. To address this, a suite of computational methods has been developed to optimize each step of the scATAC-seq workflow, from preprocessing steps such as alignment, quality control, and construction of feature-by-cell matrices to downstream analyses including dimensionality reduction, clustering, identification of differentially accessible regions (DARs), trajectory inference, and integration with transcriptomic data. Together, these approaches enhance the interpretability of sparse chromatin landscapes and facilitate insights into dynamic regulatory programs. Continued innovation in experimental protocols, including spatially resolved profiling, and in analytical strategies, such as those leveraging large-scale and specialized Artificial Intelligence (AI) models, is expected to further advance our understanding of chromatin-mediated gene regulation at single-cell resolution.

This review outlines the scATAC-seq analysis workflow, highlights key computational tools and databases, and examines the underlying principles. We focus on recent advances and provide practical guidance for effective data analysis.

## Overview of scATAC-seq data analysis

As with other single-cell modalities, scATAC-seq analysis begins with the construction of a feature-by-cell matrix, where features represent genomic regions of either fixed or variable size, and entries reflect chromatin accessibility within each cell. A primary challenge in analyzing scATAC-seq data is the extreme sparsity of this matrix: it often comprises thousands to millions of features, with most entries being zero. This sparsity, combined with the complexity of the data, poses significant analytical hurdles across all scATAC-seq platforms. Another limitation is the lack of well-defined cell-type markers based on chromatin accessibility alone, which often necessitates integration with complementary datasets such as single-cell transcriptomics [single-cell RNA sequencing (scRNA-seq)] [7,8]. Accordingly, multiomics integration has become essential for accurate cell type annotation and functional interpretation.

**Figure 1:**
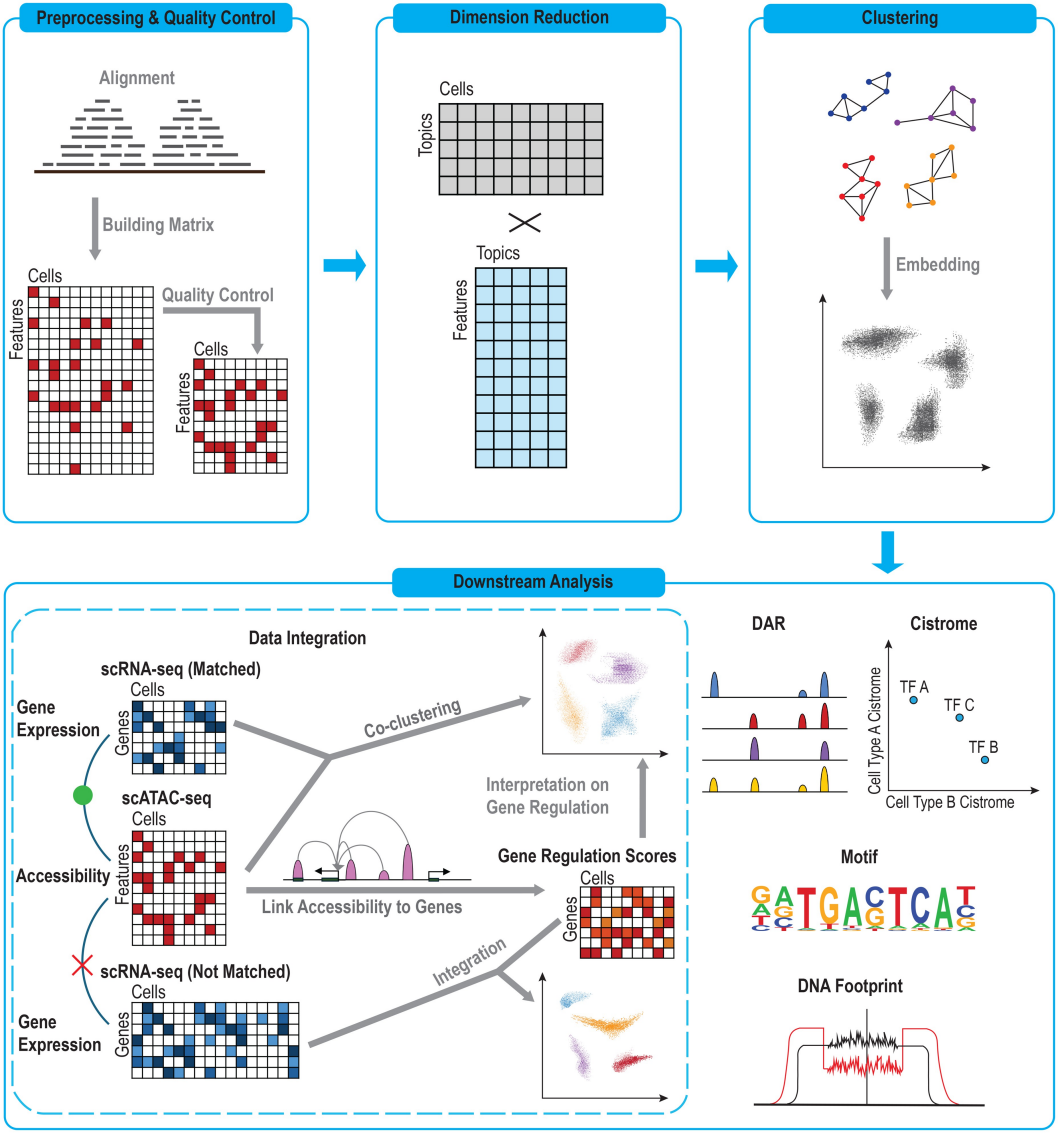
Overview of the scATAC-seq data analysis pipeline This schematic illustrates the key computational steps in scATAC-seq data analysis. Starting from raw sequencing reads, the workflow proceeds through quality control, alignment, and peak calling to generate a chromatin accessibility matrix. This matrix is subsequently normalized and reduced in dimensionality to retain key biological signals while mitigating technical noise. Downstream analyses include clustering to identify groups of cells with similar accessibility profiles and low-dimensional embedding for visualization. Cell type annotation, often aided by integration with scRNA-seq data, enables biological interpretation, while differential accessibility analysis reveals cell-type-specific regulatory elements. Additional analyses, including motif enrichment, Cistrome association, and TF footprinting, further elucidate the regulatory architecture. Integration of scATAC-seq with scRNA-seq can follow two major strategies: computing gene activity scores from chromatin accessibility profiles for alignment with expression data, or joint analysis through co-embedding when both modalities are measured in the same cells. Each computational step involves distinct challenges and is supported by specialized tools, which are reviewed in this article. scATAC-seq, single-cell Assay for Transposase-Accessible Chromatin using sequencing; scRNA-seq, single-cell RNA sequencing; DAR, differentially accessible region; TF, transcription factor.

To address these challenges, a growing suite of computational methods and tools has been developed, each requiring careful consideration depending on the analytical goals. In the following sections, we outline existing analysis packages and organize the scATAC-seq workflow into six major components: (1) preprocessing and quality control; (2) dimensionality reduction; (3) clustering of cells; (4) embedding visualization; (5) downstream analysis, including differential accessibility, cell type annotation, regulatory element inference, and trajectory analysis; and (6) integration with other assays, particularly with scRNA-seq data (**Figure 1**).

## Summary of analysis packages

The bioinformatic analysis of scATAC-seq data requires an array of computational, mathematical, and statistical tools to process, organize, visualize, and interpret high-dimensional sequencing data. To streamline this complex workflow, researchers have developed software pipelines, integrated sets of algorithms and tools executed in a predefined sequence, that serve as common entry points for data analysis (see **Table 1** for definitions of terminologies). These pipelines offer several advantages: they automate and standardize key steps, saving time and computational effort; they facilitate reproducibility; they ensure compatibility between components; and they often include user-friendly interfaces, documentation, and tutorials. Many also provide publication-ready figures and summary reports, enhancing accessibility for researchers across disciplines.

**Table 1:** Definition of terminologies

Term	Definition
Algorithm	A defined set of computational steps or rules designed to solve a specific problem or perform a particular task
Tool	A standalone software application or script that performs a specific function within a larger analytical process
Toolkit	A collection of interrelated tools developed to address a range of tasks within a particular analytical domain
Package	A bundled software resource that may include tools, libraries, and scripts, often providing a comprehensive solution for a specific type of analysis
Workflow	A high-level schematic of the analytical process, outlining the sequence of steps from data preprocessing to downstream interpretation
Pipeline	An integrated set of tools or scripts executed in a predefined sequence to automate specific stages of the analysis, ensuring efficiency and reproducibility

However, no single pipeline covers the full spectrum of scATAC-seq analyses. It is therefore critical to understand the scope, strengths, and limitations of each package, especially when integrating outputs into more specialized tools. We summarize the representative scATAC-seq analysis packages in Table S1, including: Cellranger-ATAC/ARC [9], APEC [10], Cicero [11], ArchR [12], MAESTRO [13], Signac [14], EpiScanpy [15], scATAC-pro [16], Destin [17], chromVAR [18], RA3 [19], scABC [20], scOpen [21], SnapATAC [22], cisTopic [23], Scasat [24], SCRAT [25], scATACpipe [26], and scVI/PeakVI [27]. As most of these packages are actively maintained and updated, we provide documentation links and recommend consulting the respective websites for the latest versions and feature updates (Table S1).

## Preprocessing and quality control

Preprocessing of scATAC-seq data starts with barcode-aware alignment of adapter-trimmed reads to a reference genome; many pipelines perform trimming, read filtering, and barcode correction within this alignment step. Aggregated fragments are then passed to peak callers to delineate candidate accessible regions. Only after these regions are defined are quality-control metrics such as total fragments per cell, insertion-length periodicity, transcription-start-site enrichment, and the fraction of fragments in peaks or blacklist loci calculated to exclude low-quality cells and establish the usable cell population. A feature-by-cell matrix is subsequently assembled: rows represent either data-driven peaks or predefined fixed genomic bins, and columns correspond to individual barcoded cells. Fragment counts are normalized for library size and corrected for batch or other technical effects, and data transformations such as binarization, Term Frequency-Inverse Document Frequency (TF-IDF) weighting, and optional imputation reduce sparsity and standardize the matrix, yielding an analysis-ready representation of genome-wide chromatin accessibility for downstream dimensionality reduction and clustering.

### Read preprocessing and alignment

Preprocessing tasks such as adapter trimming, barcode parsing and correction, and deduplication are performed prior to or alongside alignment, often using tools like Trimmomatic [28] or custom scripts. During this stage, cell barcodes are extracted and corrected so that each read can be associated with its cell of origin. Alignment then refers specifically to mapping sequencing reads to the reference genome. Popular aligners for short-read sequencing include Bowtie2 [29], BWA [30], Hisat2 [31], minimap2 [32], and Chromap [33]. Among these, BWA is widely adopted and integrated into pipelines such as Cellranger-ATAC or Cellranger-ARC for 10X Genomics data. Chromap, designed specifically for chromatin-profiling data, integrates alignment with adapter trimming, barcode correction, and deduplication, and has been reported to be up to 16-fold faster than BWA due to its minimizer-based strategy [33].

### Peak calling

Peak calling identifies genomic regions enriched for transposase-accessible chromatin, serving as proxies for regulatory activity. These genomic regions, or “peaks”, are detected by statistical algorithms that distinguish signal from background noise. Due to the low coverage of individual cells, peak calling is typically performed on aggregated reads from all cells to define a set of consensus peaks. These peaks form the basis of the feature-by-cell matrix used in downstream analyses. Cluster- or cell-type-specific peaks can also be identified by aggregating reads post-clustering. MACS [34], originally developed for chromatin immunoprecipitation followed by sequencing (ChIP-seq), remains the most widely used peak caller in scATAC-seq workflows due to its robustness and scalability.

### Controlling data quality

Quality control (QC) is essential for scATAC-seq analysis, with most toolkits offering built-in QC modules and visualization. Dedicated tools such as Dr.seq2 [35] also provide comprehensive QC assessments. QC is typically conducted at multiple levels: read level (*e.g.*, using FastQC [36]), bulk dataset level, single-cell level, and cluster level. Here we list the common QC criteria.

#### Total reads per cell

This metric reflects sequencing depth. Cells with very low read counts may lack informative signal, while excessively high counts may indicate doublets or technical artifacts. Thresholds are typically set based on the distribution of reads per cell.

#### Insertion length distribution

This metric reflects library quality. Insertion lengths from aligning paired-end reads should exhibit clear nucleosome periodicity, corresponding to mono- and multi-nucleosome spacing. Enrichment of short fragments is expected, as they primarily arise from accessible chromatin regions rather than nucleosome linker regions. This distribution can be assessed at the bulk or cluster level.

#### TSS enrichment

Reads are expected to accumulate at transcription start sites (TSSs) of active genes, where the chromatin is accessible. The TSS enrichment score, calculated as a signal-to-noise ratio around TSSs [37], serves as a robust QC indicator. High scores suggest good library quality, while low scores may indicate technical issues. This metric also facilitates filtering of low-quality cells at the single-cell level.

#### Fraction of fragments in peaks

This signal-to-noise ratio metric represents the proportion of reads falling within ATAC-seq peaks. Low fractions (*e.g.*, < 15%–20%) often indicate poor-quality cells. An alternative approach is to assess the fraction of fragments mapping to predefined regions of expected chromatin accessibility, such as DNase I hypersensitive sites (DHSs) aggregated from ENCODE cell lines. The ENCODE blacklist [38], which marks regions prone to artifactual signal, can also be used to exclude low-quality cells with disproportionate read mapping to these loci.

### Building the data matrix

In scATAC-seq analysis, the data matrix represents chromatin accessibility across single cells, with rows corresponding to genomic features and columns to individual barcoded cells. Unlike scRNA-seq, where the matrix captures gene expression, scATAC-seq matrices quantify accessibility, typically as read counts across genomic regions. The raw read count matrix will be further transformed (see the “Transforming data” section). Additional matrix types can be generated depending on the analytical objective, including gene activity scores, motif occurrences, k-mer frequencies, or base-pair resolution insertion profiles [39].

#### Peak-based matrices

Quantify chromatin accessibility as read coverage across defined genomic regions per cell, typically identified by peak calling or derived from external annotations such as ENCODE DHSs or candidate *cis*-regulatory elements (cCREs) [40]. Promoter regions or gene bodies may also be used when focusing on gene-level regulation. However, predefined regions may miss rare, cell-type-specific elements. To address this, cluster-specific peaks can be identified post hoc and incorporated into the feature set to enhance resolution.

#### Bin-based matrices

Segment the genome into uniformly sized, non-overlapping bins and quantify read coverage per bin per cell. This approach avoids reliance on predefined regions and can capture accessibility in rare or unannotated elements. However, bin size selection is critical: smaller bins increase resolution but also sparsity and computational load, while larger bins may obscure fine-scale regulatory features. Tools such as ArchR [12] implement this strategy using 500-bp bins.

Beyond genomic region-based approaches, other feature matrices leveraging base-pair resolution have also been developed. ChromVAR [18] constructs motif-by-cell matrices by mapping reads to transcription factor binding motifs, enabling the inference of variability in motif accessibility across cells. BROCKMAN [41] generates k-mer-by-cell matrices by counting the occurrence of short nucleotide sequences (k-mers) in each cell. The choice of k affects sensitivity and computational cost, with shorter k-mers offering finer resolution but increasing complexity, while longer k-mers reduce sensitivity.

### Removing biases

Mitigating technical biases in scATAC-seq is essential to ensure that downstream analyses reflect true biological variation. Key sources of bias include multiplets, batch effects, and sequence-specific cleavage preferences inherent to the Tn5 transposase employed in scATAC-seq.

#### Detecting and removing multiplets

Multiplets, instances where two or more cells (or more precisely in most of scATAC-seq experiments, nuclei) are captured together, can distort clustering, annotation, and differential analyses by producing artificial hybrid profiles. Detection strategies fall into two main categories: simulation-based and count-based. Tools like Scrublet [42], scDblFinder [43], and ArchR [12] simulate doublets and identify cells resembling these synthetic profiles. In contrast, AMULET [44] applies a count-based approach, flagging cells with read counts inconsistent with diploid expectations across accessible regions.

#### Correcting batch effects

Batch effects arise from technical variation across replicates, such as differences in library preparation, sequencing runs, or sample handling [45]. These artifacts can mask biological signals and hinder cross-sample comparisons. Although careful experimental design is ideal, computational methods are widely used for correction. Approaches originally developed for scRNA-seq, including Harmony [46], ComBat [47], CCA [48], MNN [49], deepMNN [50], and fastMNN [49], are increasingly adapted for scATAC-seq. Additionally, scATAC-seq-specific methods such as PeakVI [27] and BAVARIA [51] use variational autoencoders (VAEs) [52] to model and correct signal distributions across cells and batches.

#### Correcting cleavage bias

The Tn5 transposase exhibits sequence preference, leading to cleavage bias in ATAC-seq data [53]. This issue is particularly problematic in sparse single-cell datasets. SELMA [54] addresses this by modeling intrinsic cleavage bias using a k-mer-based approach with simplex encoding. It leverages mitochondrial DNA reads, assumed to be constitutively accessible, to estimate bias, even when only a small fraction of such reads is available. SELMA then assigns a Peak Bias Score (PBS) to each region and applies a weighting function, derived from PBS percentiles and optimized for consistency with scRNA-seq-based cell classification, to adjust the peak-by-cell matrix prior to downstream analyses.

### Transforming data

Unlike scRNA-seq, where features are defined as genes, scATAC-seq matrices use a broader range of genomic features, including peaks, bins, or motifs. To facilitate downstream analyses such as clustering and dimensionality reduction, various data transformations are typically applied to the raw read count matrix.

#### Binarization

Binarization converts counts into binary values representing accessibility, with “1” for accessible and “0” for inaccessible regions, based on the assumption that each site can only be present in a limited number (*e.g.*, twice in a diploid genome). This simplifies calculations and reduces the impact of amplification noise, but it also discards quantitative information and overlooks allele-specific signals or copy number variation, which may be biologically relevant in contexts such as cancer.

#### TF-IDF transformation

Originally developed for text mining, the TF-IDF method is widely used in scATAC-seq to weight features by their informativeness across cells. Here, cells are treated as documents, and genomic regions (*e.g.*, peaks or bins) as terms. TF measures the frequency of a region within a cell, while IDF downweighs regions found commonly in many cells. The product of TF and IDF highlights rare but informative features, improving the signal for downstream dimensionality reduction and clustering.

#### Imputation of missing data

scATAC-seq data are inherently sparse due to limited library complexity, which constrains the total number of unique fragments that can be recovered from a single cell. Although newer protocols such as ISSAAC-seq [55] have improved coverage, the number of observed fragments remains far below the number of accessible sites per cell type [56]. Imputation methods aim to recover missing signals by borrowing information from similar cells, enhancing data completeness and interpretability. The utility of imputation remains debated [57,58], but when applied carefully, it can improve downstream analysis. Simple approaches like k-nearest neighbors (kNN) replace missing values with averages from neighboring cells but are sensitive to parameter choice and outliers. More sophisticated methods include scOpen [21], using non-negative matrix factorization (NMF) to decompose the matrix into lower-dimensional latent factors, and MAGIC [59], using diffusion on a cell similarity graph to impute values. While NMF captures broad structure and MAGIC effectively models local similarity, both approaches can be computationally intensive and may introduce smoothing artifacts if not carefully tuned.

## Dimensionality reduction

As in other single-cell assays, the feature space of scATAC-seq data is inherently large, often more so than scRNA-seq, with matrix dimensionality ranging from thousands to millions of features depending on the representation used (*e.g.*, peak- *vs.* bin-based). Dimensionality reduction aims to project high-dimensional data into a lower-dimensional space while preserving essential biological variation and minimizing noise, redundancy, and technical artifacts. In scATAC-seq, this step is critical for downstream tasks such as clustering, visualization, and trajectory inference. Common approaches include general-purpose methods like principal component analysis (PCA) and singular value decomposition (SVD), as well as topic modeling techniques such as latent semantic indexing (LSI), latent Dirichlet allocation (LDA), NMF, and multidimensional scaling (MDS). Many of these methods are also employed in related tasks, including imputation, transformation, and multiomics integration.

### PCA and SVD

PCA and SVD are classical linear techniques for dimensionality reduction that aim to capture the most meaningful variation in high-dimensional data. PCA identifies orthogonal axes (principal components) along which variance is maximized, projecting data into a lower-dimensional space defined by these components. SVD, a related matrix factorization method, decomposes the original matrix into orthogonal left and right singular vectors, with singular values reflecting the variance captured along each axis. Dimensionality is reduced by retaining only components corresponding to the largest singular values.

While PCA and SVD are widely used in scRNA-seq analysis, their direct application to scATAC-seq is limited by the higher dimensionality and extreme sparsity of accessibility matrices. Consequently, applying PCA or SVD directly to raw count matrices is generally ineffective. Instead, these techniques are tailored for scATAC-seq by operating on transformed data, such as SVD applied to TF-IDF-normalized binarized matrices, as employed in LSI, detailed below.

### LSI, LDA, and NMF

To address the sparsity and nonlinearity of scATAC-seq data, topic modeling techniques originally developed for natural language processing have been adapted for dimensionality reduction. Methods such as LSI [60], LDA [61,62], and NMF [63] model relationships between features (*e.g.*, peaks or bins) and cells analogously to how documents relate to words and topics in text analysis.

LSI applies SVD to a TF-IDF-transformed matrix, identifying latent components that capture co-accessibility patterns. It is widely used due to its simplicity and computational efficiency and is implemented in tools such as Signac [14], ArchR [12], and EpiScanpy [15]. ArchR, for example, employs an iterative LSI strategy: it begins with a subset of highly variable features and cells, performs SVD, projects remaining cells into the reduced space, and refines feature selection and dimensionality iteratively to improve clustering resolution.

LDA is a generative probabilistic model that infers two distributions: topic-cell and region-topic. Each cell is modeled as a mixture of latent topics, and each topic as a distribution over accessible regions. Originally developed for population genetics [61] and text mining [62], LDA has been applied to scATAC-seq in tools such as cisTopic [23], where topics correspond to regulatory programs across cell populations.

NMF factorizes a non-negative data matrix, such as a TF-IDF-transformed accessibility matrix, into two lower-dimensional matrices representing term-by-topic and topic-by-cell relationships. By reducing the number of latent topics relative to the original features, NMF achieves dimensionality reduction while preserving biological interpretability. NMF is implemented in scOpen [21] and is also used in some imputation workflows.

### MDS and diffusion maps

MDS [64], implemented in Scasat [24], is a classical technique for reducing dimensionality by preserving pairwise distances between data points in a lower-dimensional space. Similarly, SnapATAC [22] applies diffusion maps, which model local data structure as a diffusion process on a graph, effectively capturing cell–cell relationships. Both approaches require constructing similarity matrices commonly using the Jaccard distance, which compares the overlap between sets of accessible features (*e.g.*, peaks or bins) across cells. The Jaccard distance is defined as the size of the intersection divided by the union of two sets, ranging from 0 (no overlap) to 1 (identical sets).

Regardless of the method used, the choice of the number of dimensions is a tunable parameter, whether principal components in PCA, singular values in SVD, latent topics in LSI, LDA, or NMF, or output dimensions in MDS or diffusion maps. This value is often selected using heuristics such as the elbow method, which evaluates the trade-off between model complexity and retained variance or reconstruction error.

## Clustering of cells

A central aim of scATAC-seq analysis is to identify cell populations with distinct chromatin accessibility profiles, thereby revealing regulatory heterogeneity within complex tissues. Clustering is typically performed on a reduced-dimensional representation of the data, which emphasizes informative features while mitigating noise and computational burden. In this section, we describe conventional clustering techniques, including k-means, hierarchical, and density-based methods, before introducing graph-based algorithms. The latter have been deemed more suitable approaches for single-cell analyses, due to their scalability and adeptness at capturing complex cellular relationships.

### K-means/medoids clustering

K-means, a centroid-based algorithm, partitions cells by minimizing the sum of squared distances to cluster centroids. It is computationally efficient but assumes linear separability and is sensitive to outliers. In contrast, k-medoids represents each cluster by actual data points (medoids), offering greater robustness to noise and non-linear boundaries. Consequently, k-medoids is preferred in scATAC-seq applications where data often lack linear separability; tools such as scABC [20] and Scasat [24] adopt this approach. However, both methods require predefining the number of clusters (k), are sensitive to initialization, and exhibit suboptimal performance on non-spherical structures prevalent in scATAC-seq data.

### Hierarchical clustering

Hierarchical clustering builds a tree-like structure by iteratively merging cells or clusters based on their pairwise similarity, starting with each cell as an individual cluster. The resulting dendrogram captures nested relationships among cells and can be cut at a chosen threshold to define discrete clusters. While less commonly used in scATAC-seq than graph-based approaches, hierarchical clustering is implemented in tools such as cisTopic, where it is applied to topic-by-cell matrices to reveal cell groupings.

### Density clustering

Density-based clustering identifies groups of cells concentrated in high-density regions of the feature space without requiring predefined cluster numbers. In scATAC-seq analysis, it is typically applied to the dimensionality-reduced feature matrix to mitigate the impact of sparsity, rather than to visualization-oriented embeddings such as *t*-Distributed Stochastic Neighbor Embedding (*t*-SNE) or Uniform Manifold Approximation and Projection (UMAP), which do not preserve density information. The most widely used algorithm in this category is Density-Based Spatial Clustering of Applications with Noise (DBSCAN) [65], implemented in tools such as MAESTRO [13] and SCRAT. DBSCAN is effective at detecting clusters of varying shapes and separating noise, though its performance is contingent on parameter optimization, particularly in datasets exhibiting uneven density.

### Graph-based clustering

Graph-based methods have emerged as prominent approaches for clustering in single-cell analysis due to their ability to capture complex relationships in high-dimensional data. These methods begin by constructing a kNN graph from the dimensionality-reduced matrix, where each cell is a node connected to its k most similar neighbors based on a distance metric, typically Jaccard, Euclidean, Manhattan, or cosine similarity, in scATAC-seq analysis. The choice of k influences graph connectivity: higher values yield denser graphs, while lower values emphasize local structure. Once the kNN graph is constructed, community detection algorithms such as Louvain and Leiden are applied to identify discrete cell clusters.

#### Louvain clustering

The Louvain algorithm [66] detects communities by maximizing modularity, a metric that evaluates how densely connected nodes are within clusters compared to between clusters. It is widely used in scATAC-seq to group cells with similar chromatin accessibility profiles. The Smart Local Moving (SLM) algorithm [67], an enhanced variant of Louvain, refines modularity optimization through local node movements, improving cluster resolution. SLM is implemented in packages such as Signac [14].

#### Leiden clustering

The Leiden algorithm [68] further improves upon Louvain and SLM by optimizing a cost function called stability, which measures the persistence of clusters under perturbation. It offers improved robustness and reproducibility, especially in noisy datasets. Leiden clustering is supported in packages such as EpiScanpy [15], and is increasingly preferred for its scalability and consistency across diverse single-cell applications.

## Embedding visualization

Low-dimensional embeddings are commonly used to visualize scATAC-seq data in two or three dimensions, enabling intuitive exploration of cell-to-cell variation. While this is technically a form of dimensionality reduction, the goal is specifically to preserve local and global structure for visualization, rather than for downstream analysis. Although methods like PCA can be used, non-linear techniques such as *t*-SNE [69] and UMAP [70] are preferred for their ability to capture complex relationships in 2D or 3D space. Importantly, these embeddings are typically not used as input for clustering or other downstream analyses, as they are optimized for display rather than feature representation.

### *t*-SNE

*t*-SNE [69] is a widely used method for visualizing high-dimensional single-cell data, including scATAC-seq, in two or three dimensions. It aims to preserve local structure by mapping similar cells close together in the embedding space, making it particularly effective for revealing discrete clusters.

The algorithm constructs a probability distribution over cell pairs in the high-dimensional space, typically based on a similarity measure such as a Gaussian kernel applied to the kNN graph, and seeks a low-dimensional representation with a matching distribution. It then minimizes the Kullback-Leibler (KL) divergence between the two distributions using gradient descent, iteratively adjusting coordinates until convergence.

The resulting 2D or 3D embedding enables intuitive visualization, where cell clusters appear as distinct groupings and outliers are spatially separated. While *t*-SNE excels at preserving local structure, it does not reliably capture global relationships and is sensitive to parameter choices, such as perplexity and initialization.

### UMAP

UMAP [70] is a non-linear dimensionality reduction technique that, like *t*-SNE, constructs a kNN graph to model relationships between cells. It aims to preserve both local and global structures by minimizing the cross-entropy between the high- and low-dimensional representations [71], yielding a layout well-suited for visualization.

Compared to *t*-SNE, UMAP is computationally more efficient and scales better to large datasets. It also tends to better preserve global structure, resulting in more continuous trajectories and spatially coherent clusters. The method is relatively robust to parameter changes, with the parameter for the number of neighbors to be considered serving as a key control for the balance between local and global preservation; lower values emphasize local relationships, while higher values retain broader structure.

Due to its scalability, speed, and flexibility, UMAP has become a widely used embedding method in scATAC-seq and other single-cell applications.

## Downstream analysis

Downstream analyses in scATAC-seq are essential for interpreting the regulatory landscape and linking chromatin accessibility to gene function and cell identity. These analyses provide insights into gene regulation, lineage trajectories, and *cis*-regulatory architecture. Key downstream tasks include:

1. Gene Regulatory Scoring: Quantifying the relationship between chromatin accessibility and nearby gene activity.
2. Cell Type Annotation: Assigning biological meaning to cell clusters by integrating chromatin profiles with known marker genes or matched transcriptomic data.
3. Differential Accessibility Analysis: Identifying DARs between cell types or states to uncover regulatory elements driving cell identity.
4. Regulatory Element Inference: Discovering transcription factor motifs, Cistromes, and footprinting signatures within DARs to reveal upstream regulators.
5. Trajectory Inference: Reconstructing developmental or dynamic epigenetic trajectories from chromatin accessibility patterns.
6. Co-accessibility Analysis: Linking distal regulatory elements (*e.g.*, enhancers) to target genes by identifying statistically co-accessible regions across single cells.

### Gene regulatory scoring

Gene regulation scores quantify the contribution of chromatin accessibility to gene activity at the single-cell level, enabling the conversion of peak- or bin-level accessibility data into a gene-by-cell matrix. This transformation facilitates biological interpretation and allows for the application of analytical frameworks developed for scRNA-seq, including differential gene activation and multiomics integration.

Several strategies have been developed to estimate gene regulation scores from scATAC-seq data. A basic approach involves counting reads within promoter regions, under the assumption that promoter accessibility correlates with transcriptional activity. More refined methods incorporate reads from both promoters and gene bodies, or apply smoothing techniques across gene regions, as implemented in tools such as Signac [14] and SnapATAC [22].

Advanced methods, such as those used in MAESTRO [13] and ArchR [12], calculate gene scores as a weighted sum of chromatin accessibility signals from regions surrounding the TSSs, including distal regulatory elements. Weights are typically assigned based on genomic distance, often using exponential decay functions. In a comprehensive benchmarking study [12], the authors compared 56 approaches and found that models integrating promoter, gene body, and distal element accessibility, with distance-based weighting, provided the most accurate reflection of gene regulatory activity.

### Cell type annotation

Accurate cell type annotation is essential for interpreting scATAC-seq data, enabling the identification of cell-type-specific regulatory landscapes, improving reproducibility, and uncovering novel cellular subpopulations. Two main strategies are commonly used: annotation based on gene regulatory scores derived from scATAC-seq, and label transfer from reference gene expression datasets such as scRNA-seq.

The first approach mirrors strategies from scRNA-seq, where differentially activated genes (based on regulatory scores) are matched to known marker genes from curated databases, including CellMarker [72], PanglaoDB [73], the Human Cell Atlas [74], and Tabula Muris [75]. However, regulatory scores from scATAC-seq do not always correlate positively with gene expression, limiting the accuracy of marker-based annotation when used in isolation.

Given the increasing prevalence of multiomics designs, a more robust strategy involves label transfer from matched scRNA-seq datasets. This approach aligns chromatin accessibility profiles with annotated transcriptomic references using integration methods described in the “Multiomics integration” section. Comparative evaluations [76] have demonstrated that label transfer improves annotation accuracy and consistency, making it the preferred method when both data types are available.

### Differential accessibility analysis

Identifying DARs between cell types or clusters is central to characterizing cell-type-specific regulatory landscapes in scATAC-seq. By comparing chromatin accessibility profiles across groups of cells, DARs highlight genomic regions, typically peaks or bins, that are more accessible in one group than another.

Unlike bulk assays, where traditional tools like DESeq2 [77], edgeR [78], or Limma [79] are used, scATAC-seq presents distinct statistical challenges due to its high sparsity and large sample sizes (*i.e*., many cells). Treating each cell as an independent observation violates assumptions of conventional methods. Instead, non-parametric tests, particularly the Wilcoxon rank-sum test, have shown better performance for identifying DARs in this context [80]. This approach is now the default in several widely used scATAC-seq toolkits, including ArchR [12], Signac [14], and MAESTRO [13].

### Discovering key regulatory events in DARs

DARs provide a valuable entry point for identifying key transcription factors (TFs) and regulatory programs active across cell types or states. By linking DARs to transcription factor binding motifs [81], Cistromes [82], and footprinting patterns [83], researchers can infer cell-type-specific regulatory mechanisms underlying differentiation and lineage specification. Together, these approaches enable comprehensive characterization of TF activity and regulatory circuits in single-cell epigenomic landscapes.

#### Motif enrichment analysis

TF motif enrichment can be assessed in DARs using traditional tools developed for bulk data, including HOMER [84] and MEME [85], with motif libraries such as JASPAR [86] and Cistrome [82]. These analyses scan DAR sequences for overrepresented motifs corresponding to candidate TFs. ChromVAR supports single-cell resolution by generating motif-by-cell matrices and computing deviations in motif accessibility across the entire cell population, enabling direct identification of differentially enriched motifs [18].

#### Cistrome enrichment analysis

The Cistrome database [82] aggregates genome-wide profiles and chromatin accessibility data from ChIP-seq and ATAC-seq experiments across diverse cell types. Tools such as MAESTRO [13] leverage this resource to link DARs with candidate TFs by querying for enriched overlaps using GIGGLE [87], a genomic interval search tool. GIGGLE calculates enrichment statistics and generates a ranked list of candidate TFs for each cell type or cluster. These can be further prioritized by weighting with gene regulation scores derived from scATAC-seq.

#### Footprinting analysis

TF binding induces localized changes in chromatin structure, such as nucleosome displacement and recruitment of chromatin remodelers, that alter DNA accessibility. These changes create characteristic “footprints”, observed as localized depletions of transposase insertions at motif sites in ATAC-seq data [88]. In scATAC-seq, footprinting analysis enables the inference of direct TF–DNA interactions in a cell-type-specific manner. To identify footprints, the genomic locations of known TF motifs are mapped within accessible regions, and aggregate insertion profiles are analyzed around these sites. Differences in footprint depth across cell clusters can reveal dynamic TF binding activity. Footprinting functions are supported in ArchR [12], scATACpipe [26] (built on ArchR), and scATAC-pro [16], which integrates the HINT-ATAC algorithm [89]. PRINT [39], a deep learning–based tool, has been introduced to detect multi-scale TF footprints with improved resolution. PRINT corrects for Tn5 sequence bias and estimates footprint scores at base-pair resolution using adaptive kernels, enabling the detection of DNA-binding proteins with diverse binding site characteristics across enhancers and promoters.

### Trajectory and pseudotime analyses

Trajectory analysis in scATAC-seq aims to reconstruct dynamic biological processes, such as development, differentiation, or response to stimuli, by modeling the progression of chromatin accessibility states across single cells. Unlike scRNA-seq, which infers pseudotime from gene expression, scATAC-seq trajectory analysis captures changes in regulatory landscape, offering direct insight into the epigenetic mechanisms driving cell state transitions. Cells are ordered along a pseudotime axis based on accessibility profile similarity, enabling the identification of regulatory elements and TFs associated with specific transitions. This approach can also reveal intermediate or transient cell states not readily apparent from transcriptomic data. Several trajectory inference tools originally developed for scRNA-seq are adaptable to scATAC-seq. Monocle [90], included in pipelines such as APEC [10], Cicero [11], ArchR [12], and Signac [14], remains widely used. Slingshot [91], incorporated into RA3 [19], offers an alternative lineage inference framework. Additionally, STREAM [92] can infer trajectories from k-mer-by-cell matrices generated by chromVAR [18], enabling regulatory trajectory analysis based on sequence features.

### Co-accessibility and linking accessibilities to genes

Co-accessibility analysis identifies pairs of chromatin regions, such as enhancers and promoters, that exhibit correlated accessibility across single cells, suggesting coordinated regulatory activity, often through physical proximity in chromatin loops. These correlations can help link distal regulatory elements to their target genes, enhancing interpretation of the regulatory architecture captured by scATAC-seq. Statistical models are used to compute co-accessibility scores between genomic regions, generating interaction maps that infer putative enhancer–promoter connections. Tools such as Cicero [11] and ArchR [12] implement these methods, enabling genome-wide identification of co-accessible regions and their integration into gene regulatory networks.

## Multiomics integration

Integrating scATAC-seq with complementary single-cell assays, particularly scRNA-seq, enables a more comprehensive understanding of gene regulation by linking chromatin accessibility to transcriptional output. While scRNA-seq captures gene expression profiles, scATAC-seq reveals the underlying epigenetic landscape and regulatory potential. Joint analysis of these modalities helps elucidate how chromatin state shapes cell identity and function. Integration strategies generally fall into two categories: cross-modality integration, where scRNA-seq and scATAC-seq are generated from separate but comparable cells, and multiomics profiling, where both data types are obtained simultaneously from the same cell. The choice of strategy depends on experimental design and analytical goals.

### Integrating gene regulation scores with scRNA-seq

Integration of scATAC-seq with scRNA-seq is facilitated by converting chromatin accessibility profiles into gene regulation scores, aligning the features of scATAC-seq with gene expression in scRNA-seq. This transformation bridges the modality gap, enabling the application of data integration techniques to unify the datasets into a shared latent space.

Linear integration methods such as canonical correlation analysis (CCA) [48] and mutual nearest neighbors (MNN) [49] are used in tools like Seurat [48] and Signac [14] to identify anchor pairs between gene regulation scores and gene expression profiles. These anchors guide the joint embedding of the two datasets. LIGER [93] and CoupleNMF [94] apply NMF to extract shared low-dimensional representations. However, linear models may struggle to capture complex non-linear relationships across modalities. To address this, unsupervised manifold alignment methods have been proposed. MATCHER [95] employs Gaussian process latent variable models to align modalities along a shared pseudotime axis, while SCOT [96] uses optimal transport to align datasets by minimizing differences in intra-domain distance matrices.

Deep learning-based methods have emerged as powerful tools for integration of scATAC-seq and scRNA-seq. VAEs are used in Cobolt [97], scMVP [98], and MultiVI [99] (from the scVI-tools framework) to model high-dimensional scATAC-seq and scRNA-seq jointly. scDEC [100] combines VAEs with paired generative adversarial networks (GANs) to simultaneously learn latent features and perform clustering. Transfer learning approaches such as scJoint [101] further enable label propagation from annotated scRNA-seq to scATAC-seq data by learning a shared embedding based on gene-level features.

### Co-analyzing scATAC-seq and scRNA-seq multiomics data

While gene regulation scores derived from scATAC-seq offer a bridge to scRNA-seq integration, accessibility does not always correlate directly with transcriptional activity. This disconnect limits the accuracy of integrative analyses based on separate assays. Multiomics technologies, such as 10X Genomics Multiome [102] and SHARE-seq [103], overcome this limitation by simultaneously profiling chromatin accessibility and gene expression within the same cell, enabling direct, high-resolution mapping between regulatory potential and transcriptional output.

One approach for analyzing such data is weighted nearest neighbor (WNN) analysis [104]. WNN independently processes each modality, then learns cell-specific weights reflecting the relative contribution of chromatin accessibility and gene expression. A joint WNN graph is constructed by integrating modality-specific similarity measures, enabling co-clustering and downstream analyses that account for the strengths of each data type.

Rather than differentially weighting scRNA-seq and scATAC-seq multiomics data, MIRA [105] employs a probabilistic topic modeling framework to embed both transcriptomic and epigenomic data into a shared latent space. MIRA jointly models gene expression and chromatin accessibility at individual loci, generating regulatory potential (RP) scores that quantify the contribution of accessibility to gene regulation. By leveraging shared cell barcodes, MIRA maps accessibility–transcription relationships across developmental trajectories, distinguishing between chromatin features that are coupled or decoupled from transcription. This enables reconstruction of high-resolution cell state trees and identification of key regulators driving lineage bifurcations.

### Integrating scATAC-seq with bulk-level omics data

Bulk-level omics datasets, derived from well-characterized tissues or conditions, offer deep coverage and rich annotations that complement the cellular resolution of single-cell data. Integrating scATAC-seq with bulk data enhances biological interpretation by facilitating cell type annotation, regulatory element identification, and TF activity inference. By combining the granularity of single-cell data with the depth of bulk-level resources, these integrative strategies enhance the resolution, interpretability, and functional relevance of scATAC-seq analyses.

CellWalker [106] exemplifies this approach by integrating bulk gene expression and scRNA-seq data to generate cell-type-specific expression labels. These labels are connected to scATAC-seq profiles via a similarity-based network. A diffusion process propagates information across the combined graph, producing a label-by-cell matrix for annotation and a cell-by-cell matrix for clustering.

Bulk TF ChIP-seq data can also be leveraged to infer TF activity in scATAC-seq. SCRIP [107] constructs a reference of high-quality TF binding profiles from bulk ChIP-seq datasets (*e.g.*, from the Cistrome database), and uses GIGGLE [87] to identify enriched TF binding events within single cells. It further integrates these results with scATAC-seq peaks to predict downstream target genes of active TFs.

## scATAC-seq data resources

### scATAC-seq databases

Since the introduction of scATAC-seq, the accumulation of chromatin accessibility data at single-cell resolution has driven the development of dedicated databases to manage and disseminate these datasets. Early repositories focused on general purpose single-cell data, such as the Single Cell Portal by the Broad Institute [108], CELLxGENE by CZI [109], and the Human Cell Atlas [74] data portal. As the field has progressed, more specialized platforms have emerged to support targeted research applications. For instance, scATAC-Ref [110] provides curated scATAC-seq datasets with annotated cell types across five species, while scBlood [111] offers insights into blood cell heterogeneity through chromatin accessibility profiles. These databases typically offer a searchable user interface, often include both raw and processed data, and support multiple species. By facilitating data discovery and selection, these resources enable researchers to explore chromatin accessibility across diverse biological contexts. A curated summary of scATAC-seq databases is provided in Table S2.

### Simulation of scATAC-seq data

Simulated scATAC-seq data are essential for benchmarking computational methods, allowing systematic evaluation of sensitivity, specificity, and scalability in the absence of ground truth in real datasets. Simulations also enable developers to assess method robustness under varying noise levels and experimental conditions. While many toolkits include custom simulations to demonstrate performance [23,25], independent simulation frameworks offer reproducible and unbiased platforms for testing and optimizing analysis pipelines.

SCAN-ATAC-Sim [112] generates synthetic data by down-sampling bulk ATAC-seq from annotated cell types and injecting tunable background noise, simulating diploid genomes through independent sampling of accessibility events. simATAC [113] creates realistic bin-by-cell matrices by learning key parameters, such as library size, sparsity, and read count distributions, from user-supplied scATAC-seq datasets. It then generates new data by sampling from Gaussian mixture and polynomial models to match these distributions. These simulation tools provide valuable resources for method development and validation across diverse scATAC-seq applications.

### Interactive visualization of scATAC-seq data

While many scATAC-seq toolkits provide basic visualization functions for genome browser-style or cluster-level summaries, interactive visualization platforms offer greater accessibility, particularly for researchers without extensive bioinformatics training. These tools allow users to explore chromatin accessibility profiles dynamically, facilitating data interpretation, collaboration, and dissemination both before and after publication.

Visualization platforms initially developed for scRNA-seq can frequently be adapted for scATAC-seq by replacing gene expression features with chromatin accessibility data. Essential features include metadata-driven highlighting, custom cell annotations, and web-based deployment [114]. Importantly, sustained support and ongoing development are critical to maintaining usability as datasets increase in both size and complexity.

Popular tools such as ASAP [115], the UCSC Cell Browser [116], and CZ CELLxGENE [109] have become widely adopted for hosting large-scale single-cell datasets. These platforms support interactive browsing of public repositories and allow researchers to build custom web portals for sharing their own data.

## Artificial Intelligence approaches

Recent deep learning models such as PeakVI [27], PROTRAIT [117], and LINGER [118] have advanced scATAC-seq analysis by leveraging variational inference and transformer architectures to address data sparsity and uncover regulatory signals. PeakVI is a deep variational framework that learns a latent embedding of chromatin accessibility, preserving cellular heterogeneity, correcting batch effects, and enabling single-region differential accessibility analysis for cell type annotation [27]. PROTRAIT employs a ProdDep Transformer to capture TF motif syntax, supporting chromatin accessibility prediction, single-cell embedding for cell type annotation, scATAC-seq denoising, and single-nucleotide resolution inference of TF activity [117]. LINGER introduces a continuous-learning neural framework that integrates atlas-scale external data and TF motif knowledge to infer context-specific gene regulatory networks from paired scATAC-seq and scRNA-seq data [118].

Foundation models are also beginning to transform the field. CREformer, a 3-billion-parameter multimodal transformer pre-trained on 55 billion nucleotide sequences and 165 million single-cell multiomics profiles, enables zero-shot prediction of master regulators, enhancers, gene regulatory networks, and functional variants without fine-tuning [119]. EpiFoundation [120] uses cross-modal pre-training (encoding nonzero peak signals and aligning them with matched gene expression) to learn cell representations that carry out downstream tasks such as cell type labeling, batch effect correction, and gene expression imputation. scGPT [121], a generative pre-trained transformer trained on over 33 million single-cell profiles, captures a “cellular language” model that, once fine-tuned, achieves state-of-the-art performance in cell annotation, multi-batch and multiomics integration, perturbation-response prediction, and gene network inference. More recently, ChromFound [122] was introduced as a foundation model tailored for scATAC-seq, utilizing a hybrid architecture and genome-aware tokenization to deliver robust zero-shot performance in cell representation and cross-omics prediction.

A recent illustration of how experimental and deep learning innovation can converge is the ultra-throughput, ultra-sensitive single-nucleus ATAC-seq protocol (UUATAC-seq) [123], which is able to generates a genome-wide chromatin-accessibility map for an entire species in a single day. Coupled with UUATAC-seq, the mega-task deep-learning model Nvwa *cis*-regulatory element (NvwaCE) learns higher-order “regulatory grammar” directly from sequence and predicts candidate *cis*-regulatory element (cCRE) landscapes with base-pair-level precision across multiple vertebrate species. Integrating such ultra-high-throughput assays and interpretable AI models into standard scATAC-seq pipelines could accelerate variant prioritization, cross-species comparative studies, and the clinical translation of regulatory genomics.

## Spatial applications

Recent advances in spatially resolved scATAC-seq have enabled high-resolution chromatin accessibility profiling within intact tissues. Newly emerging experimental platforms now leverage spatial barcoding strategies to achieve near single-cell resolution. For instance, microfluidic-based *in situ* deterministic barcoding can deposit DNA barcode grids directly onto tissue sections, encoding positional information at ∼ 10 µm (near single-cell) resolution [124]. Alternatively, solid-phase capture approaches use barcoded oligonucleotide arrays on slides to bind Tn5-tagmented fragments, enabling genome-wide open-chromatin profiling with spatial resolution [125]. These approaches represent an improvement over earlier methods such as sciMAP-ATAC, which employs combinatorial indexing of tissue micro-punches and is limited to ∼ 200 µm spatial resolution [126].

Concurrently, the integration of spatial epigenomics with spatial transcriptomics [127] has progressed rapidly, supported by emerging analysis tools. Multimodal protocols such as spatial-ATAC-RNA-seq enable simultaneous profiling of the chromatin accessibility and gene expression within the same tissue slice, directly linking regulatory landscapes to local transcriptional programs [128]. To analyze the resulting sparse, high-dimensional data, new computational tools have been developed. These include methods for detecting spatially variable chromatin accessibility peaks and domains (Descart [129]), as well as integrative algorithms that map dissociated scATAC-seq or multiomics datasets onto spatial coordinates (SIMO [130]). Collectively, these advances enhance the spatial epigenomics toolkit, allowing for increasingly comprehensive *in situ* reconstruction of gene regulatory programs.

## Conclusion

In conclusion, scATAC-seq is a powerful method for exploring the epigenetic landscape of individual cells and uncovering the diversity of chromatin structures and regulatory functions. Ongoing innovations in experimental protocols, including spatially resolved epigenomics, and analytical approaches, such as those employing specialized and large-scale AI models, are poised to further expand the scope and resolution of chromatin biology at the single-cell level. The analysis of scATAC-seq data involves various computational steps, including quality control, alignment, peak calling, dimensionality reduction, clustering, gene regulation scoring, cell type annotation, and multiomics data integration. A growing array of tools and algorithms support each of these tasks, though their performance varies by context, requiring informed choices based on data characteristics and study objectives. Interpreting scATAC-seq data also demands careful consideration of biological relevance and methodological assumptions. When properly executed, scATAC-seq analysis deepens our understanding of cell states, developmental trajectories, and disease mechanisms.

Despite rapid advance, key challenges remain. First, the increasing throughput of sequencing platforms, yielding more sequenced cells per sample or deeper coverage per cell, imposes growing computational demands on scATAC-seq analysis, particularly with respect to memory usage. Recent developments in computational technologies, such as leveraging disk-based strategies to mitigate memory bottlenecks (*e.g.*, Scarf [131]), show promise. Second, the field requires sustained community efforts to benchmark emerging methods for scATAC-seq analysis [132], establish standards, and provide curated test datasets. An open-access benchmarking platform could facilitate regular evaluations and competitions, enabling developers and end-users to compare algorithms on standardized datasets with known ground truth. Open-science initiatives such as the DREAM Challenge [133] and Kaggle’s multimodal single-cell integration competition [134] exemplify frameworks for testing new algorithms on standardized datasets with ground truth annotations. Finally, future advances will benefit from incorporating ATAC-seq-specific biases and features into single-cell analysis workflows. For example, Tn5 transposase cutting biases and nucleosome positioning [135] remain largely overlooked due to the sparse coverage of individual cells. As sequencing technologies evolve and data quality and coverage improve, accounting for such ATAC-seq-specific biases and features may enhance the resolution, interpretability, and biological accuracy of scATAC-seq data analysis.

## CRediT author statement

**Chenfei Wang:** Writing – original draft, Writing – review & editing. **Jiaojiao Zhou:** Investigation, Writing – original draft. **Hong Zhang:** Visualization, Writing – review & editing. **Zihan Zhuang:** Investigation, Writing – review & editing. **Gali Bai:** Conceptualization, Writing – original draft. **Ming Tang:** Conceptualization, Investigation, Writing – original draft, Writing – review & editing. **Song Liu:** Supervision, Writing – original draft, Writing – review & editing. **Tao Liu:** Conceptualization, Supervision, Visualization, Writing – original draft, Writing – review & editing. All authors have read and approved the final manuscript.

## Competing interests

The authors have declared no competing interests.

## Supplementary material

Supplementary material is available at *Genomics, Proteomics & Bioinformatics* online (https://doi.org/10.1093/gpbjnl/qzaf115).

## Supplementary Material

qzaf115_Supplementary_Data

## References

[1] Buenrostro JD , Wu B, Litzenburger UM, Ruff D, Gonzales ML, Snyder MP, et al Single-cell chromatin accessibility reveals principles of regulatory variation. Nature 2015;523:486–90. 26083756 10.1038/nature14590 PMC4685948

[2] Zhu C , Preissl S, Ren B. Single-cell multimodal omics: the power of many. Nat Methods 2020;17:11–4. 31907462 10.1038/s41592-019-0691-5

[3] Cusanovich DA , Daza R, Adey A, Pliner HA, Christiansen L, Gunderson KL, et al Multiplex single cell profiling of chromatin accessibility by combinatorial cellular indexing. Science 2015;348:910–4. 25953818 10.1126/science.aab1601 PMC4836442

[4] Sinha S , Satpathy AT, Zhou W, Ji H, Stratton JA, Jaffer A, et al Profiling chromatin accessibility at single-cell resolution. Genomics Proteomics Bioinformatics 2021;19:172–90. 33581341 10.1016/j.gpb.2020.06.010 PMC8602754

[5] Cheng S , Miao B, Li T, Zhao G, Zhang B. Review and evaluate the bioinformatics analysis strategies of ATAC-seq and CUT&Tag data. Genomics Proteomics Bioinformatics 2024;22:qzae054. 39255248 10.1093/gpbjnl/qzae054 PMC11464419

[6] Chen H , Lareau C, Andreani T, Vinyard ME, Garcia SP, Clement K, et al Assessment of computational methods for the analysis of single-cell ATAC-seq data. Genome Biol 2019;20:241. 31739806 10.1186/s13059-019-1854-5 PMC6859644

[7] Shi Q , Chen X, Zhang Z. Decoding human biology and disease using single-cell omics technologies. Genomics Proteomics Bioinformatics 2023;21:926–49. 37739168 10.1016/j.gpb.2023.06.003 PMC10928380

[8] Wang R , Peng G, Tam PPL, Jing N. Integration of computational analysis and spatial transcriptomics in single-cell studies. Genomics Proteomics Bioinformatics 2023;21:13–23. 35901961 10.1016/j.gpb.2022.06.006 PMC10372908

[9] Satpathy AT , Granja JM, Yost KE, Qi Y, Meschi F, McDermott GP, et al Massively parallel single-cell chromatin landscapes of human immune cell development and intratumoral T cell exhaustion. Nat Biotechnol 2019;37:925–36. 31375813 10.1038/s41587-019-0206-z PMC7299161

[10] Li B , Li Y, Li K, Zhu L, Yu Q, Cai P, et al APEC: an accesson-based method for single-cell chromatin accessibility analysis. Genome Biol 2020;21:116. 32398051 10.1186/s13059-020-02034-y PMC7218568

[11] Pliner HA , Packer JS, McFaline-Figueroa JL, Cusanovich DA, Daza RM, Aghamirzaie D, et al Cicero predicts *cis*-regulatory DNA interactions from single-cell chromatin accessibility data. Mol Cell 2018;71:858–71.e8. 30078726 10.1016/j.molcel.2018.06.044 PMC6582963

[12] Granja JM , Corces MR, Pierce SE, Bagdatli ST, Choudhry H, Chang HY, et al ArchR is a scalable software package for integrative single-cell chromatin accessibility analysis. Nat Genet 2021;53:403–11. 33633365 10.1038/s41588-021-00790-6 PMC8012210

[13] Wang C , Sun D, Huang X, Wan C, Li Z, Han Y, et al Integrative analyses of single-cell transcriptome and regulome using MAESTRO. Genome Biol 2020;21:198. 32767996 10.1186/s13059-020-02116-x PMC7412809

[14] Stuart T , Srivastava A, Madad S, Lareau CA, Satija R. Single-cell chromatin state analysis with Signac. Nat Methods 2021;18:1333–41. 34725479 10.1038/s41592-021-01282-5 PMC9255697

[15] Danese A , Richter ML, Chaichoompu K, Fischer DS, Theis FJ, Colome-Tatche M. EpiScanpy: integrated single-cell epigenomic analysis. Nat Commun 2021;12:5228. 34471111 10.1038/s41467-021-25131-3 PMC8410937

[16] Yu W , Uzun Y, Zhu Q, Chen C, Tan K. scATAC-pro: a comprehensive workbench for single-cell chromatin accessibility sequencing data. Genome Biol 2020;21:94. 32312293 10.1186/s13059-020-02008-0 PMC7169039

[17] Urrutia E , Chen L, Zhou H, Jiang Y. Destin: toolkit for single-cell analysis of chromatin accessibility. Bioinformatics 2019;35:3818–20. 30821321 10.1093/bioinformatics/btz141 PMC6761983

[18] Schep AN , Wu B, Buenrostro JD, Greenleaf WJ. chromVAR: inferring transcription-factor-associated accessibility from single-cell epigenomic data. Nat Methods 2017;14:975–8. 28825706 10.1038/nmeth.4401 PMC5623146

[19] Chen S , Yan G, Zhang W, Li J, Jiang R, Lin Z. RA3 is a reference-guided approach for epigenetic characterization of single cells. Nat Commun 2021;12:2177. 33846355 10.1038/s41467-021-22495-4 PMC8041798

[20] Zamanighomi M , Lin Z, Daley T, Chen X, Duren Z, Schep A, et al Unsupervised clustering and epigenetic classification of single cells. Nat Commun 2018;9:2410. 29925875 10.1038/s41467-018-04629-3 PMC6010417

[21] Li Z , Kuppe C, Ziegler S, Cheng M, Kabgani N, Menzel S, et al Chromatin-accessibility estimation from single-cell ATAC-seq data with scOpen. Nat Commun 2021;12:6386. 34737275 10.1038/s41467-021-26530-2 PMC8568974

[22] Fang R , Preissl S, Li Y, Hou X, Lucero J, Wang X, et al Comprehensive analysis of single cell ATAC-seq data with SnapATAC. Nat Commun 2021;12:1337. 33637727 10.1038/s41467-021-21583-9 PMC7910485

[23] Bravo Gonzalez-Blas C , Minnoye L, Papasokrati D, Aibar S, Hulselmans G, Christiaens V, et al cisTopic: *cis*-regulatory topic modeling on single-cell ATAC-seq data. Nat Methods 2019;16:397–400. 30962623 10.1038/s41592-019-0367-1 PMC6517279

[24] Baker SM , Rogerson C, Hayes A, Sharrocks AD, Rattray M. Classifying cells with Scasat, a single-cell ATAC-seq analysis tool. Nucleic Acids Res 2019;47:e10. 30335168 10.1093/nar/gky950 PMC6344856

[25] Ji Z , Zhou W, Ji H. Single-cell regulome data analysis by SCRAT. Bioinformatics 2017;33:2930–2. 28505247 10.1093/bioinformatics/btx315 PMC5870556

[26] Hu K , Liu H, Lawson ND, Zhu LJ. scATACpipe: a nextflow pipeline for comprehensive and reproducible analyses of single cell ATAC-seq data. Front Cell Dev Biol 2022;10:981859. 36238687 10.3389/fcell.2022.981859 PMC9551270

[27] Ashuach T , Reidenbach DA, Gayoso A, Yosef N. PeakVI: a deep generative model for single-cell chromatin accessibility analysis. Cell Rep Methods 2022;2:100182. 35475224 10.1016/j.crmeth.2022.100182 PMC9017241

[28] Bolger AM , Lohse M, Usadel B. Trimmomatic: a flexible trimmer for Illumina sequence data. Bioinformatics 2014;30:2114–20. 24695404 10.1093/bioinformatics/btu170 PMC4103590

[29] Langmead B , Salzberg SL. Fast gapped-read alignment with Bowtie 2. Nat Methods 2012;9:357–9. 22388286 10.1038/nmeth.1923 PMC3322381

[30] Li H , Durbin R. Fast and accurate short read alignment with Burrows-Wheeler transform. Bioinformatics 2009;25:1754–60. 19451168 10.1093/bioinformatics/btp324 PMC2705234

[31] Kim D , Paggi JM, Park C, Bennett C, Salzberg SL. Graph-based genome alignment and genotyping with HISAT2 and HISAT-genotype. Nat Biotechnol 2019;37:907–15. 31375807 10.1038/s41587-019-0201-4 PMC7605509

[32] Li H. Minimap2: pairwise alignment for nucleotide sequences. Bioinformatics 2018;34:3094–100. 29750242 10.1093/bioinformatics/bty191 PMC6137996

[33] Zhang H , Song L, Wang X, Cheng H, Wang C, Meyer CA, et al Fast alignment and preprocessing of chromatin profiles with Chromap. Nat Commun 2021;12:6566. 34772935 10.1038/s41467-021-26865-w PMC8589834

[34] Zhang Y , Liu T, Meyer CA, Eeckhoute J, Johnson DS, Bernstein BE, et al Model-based analysis of ChIP-Seq (MACS). Genome Biol 2008;9:R137. 18798982 10.1186/gb-2008-9-9-r137 PMC2592715

[35] Zhao C , Hu S, Huo X, Zhang Y. Drseq2: a quality control and analysis pipeline for parallel single cell transcriptome and epigenome data. PLoS One 2017;12:e0180583. 28671995 10.1371/journal.pone.0180583 PMC5495495

[36] FastQC: a quality control tool for high throughput sequence data. Babraham Bioinformatics. https://www.bioinformatics.babraham.ac.uk/projects/fastqc/ (11/18/2025 last accessed).

[37] Grandi FC , Modi H, Kampman L, Corces MR. Chromatin accessibility profiling by ATAC-seq. Nat Protoc 2022;17:1518–52. 35478247 10.1038/s41596-022-00692-9 PMC9189070

[38] Amemiya HM , Kundaje A, Boyle AP. The ENCODE blacklist: identification of problematic regions of the genome. Sci Rep 2019;9:9354. 31249361 10.1038/s41598-019-45839-z PMC6597582

[39] Hu Y , Ma S, Kartha VK, Duarte FM, Horlbeck M, Zhang R, et al Single-cell multi-scale footprinting reveals the modular organization of DNA regulatory elements. bioRxiv 2023;533945.

[40] ENCODE Project Consortium, Moore JE, Purcaro MJ, Pratt HE, Epstein CB, Shoresh N, et al Expanded encyclopaedias of DNA elements in the human and mouse genomes. Nature 2020;583:699–710. 32728249 10.1038/s41586-020-2493-4 PMC7410828

[41] de Boer CG , Regev A. BROCKMAN: deciphering variance in epigenomic regulators by k-mer factorization. BMC Bioinformatics 2018;19:253. 29970004 10.1186/s12859-018-2255-6 PMC6029352

[42] Wolock SL , Lopez R, Klein AM. Scrublet: computational identification of cell doublets in single-cell transcriptomic data. Cell Syst 2019;8:281–91.e9. 30954476 10.1016/j.cels.2018.11.005 PMC6625319

[43] Germain PL , Lun A, Garcia Meixide C, Macnair W, Robinson MD. Doublet identification in single-cell sequencing data using scDblFinder. F1000Res 2021;10:979. 35814628 10.12688/f1000research.73600.1 PMC9204188

[44] Thibodeau A , Eroglu A, McGinnis CS, Lawlor N, Nehar-Belaid D, Kursawe R, et al AMULET: a novel read count-based method for effective multiplet detection from single nucleus ATAC-seq data. Genome Biol 2021;22:252. 34465366 10.1186/s13059-021-02469-x PMC8408950

[45] Leek JT , Scharpf RB, Bravo HC, Simcha D, Langmead B, Johnson WE, et al Tackling the widespread and critical impact of batch effects in high-throughput data. Nat Rev Genet 2010;11:733–9. 20838408 10.1038/nrg2825 PMC3880143

[46] Korsunsky I , Millard N, Fan J, Slowikowski K, Zhang F, Wei K, et al Fast, sensitive and accurate integration of single-cell data with Harmony. Nat Methods 2019;16:1289–96. 31740819 10.1038/s41592-019-0619-0 PMC6884693

[47] Buttner M , Miao Z, Wolf FA, Teichmann SA, Theis FJ. A test metric for assessing single-cell RNA-seq batch correction. Nat Methods 2019;16:43–9. 30573817 10.1038/s41592-018-0254-1

[48] Butler A , Hoffman P, Smibert P, Papalexi E, Satija R. Integrating single-cell transcriptomic data across different conditions, technologies, and species. Nat Biotechnol 2018;36:411–20. 29608179 10.1038/nbt.4096 PMC6700744

[49] Haghverdi L , Lun ATL, Morgan MD, Marioni JC. Batch effects in single-cell RNA-sequencing data are corrected by matching mutual nearest neighbors. Nat Biotechnol 2018;36:421–7. 29608177 10.1038/nbt.4091 PMC6152897

[50] Zou B , Zhang T, Zhou R, Jiang X, Yang H, Jin X, et al deepMNN: deep learning-based single-cell RNA sequencing data batch correction using mutual nearest neighbors. Front Genet 2021;12:708981. 34447413 10.3389/fgene.2021.708981 PMC8383340

[51] Kopp W , Akalin A, Ohler U. Simultaneous dimensionality reduction and integration for single-cell ATAC-seq data using deep learning. Nat Mach Intell 2022;4:162–8.

[52] Gronbech CH , Vording MF, Timshel PN, Sonderby CK, Pers TH, Winther O. scVAE: variational auto-encoders for single-cell gene expression data. Bioinformatics 2020;36:4415–22. 32415966 10.1093/bioinformatics/btaa293

[53] Meyer CA , Liu XS. Identifying and mitigating bias in next-generation sequencing methods for chromatin biology. Nat Rev Genet 2014;15:709–21. 25223782 10.1038/nrg3788 PMC4473780

[54] Hu SS , Liu L, Li Q, Ma W, Guertin MJ, Meyer CA, et al Intrinsic bias estimation for improved analysis of bulk and single-cell chromatin accessibility profiles using SELMA. Nat Commun 2022;13:5533. 36130957 10.1038/s41467-022-33194-z PMC9492688

[55] Xu W , Yang W, Zhang Y, Chen Y, Hong N, Zhang Q, et al ISSAAC-seq enables sensitive and flexible multimodal profiling of chromatin accessibility and gene expression in single cells. Nat Methods 2022;19:1243–9. 36109677 10.1038/s41592-022-01601-4

[56] Thurman RE , Rynes E, Humbert R, Vierstra J, Maurano MT, Haugen E, et al The accessible chromatin landscape of the human genome. Nature 2012;489:75–82. 22955617 10.1038/nature11232 PMC3721348

[57] Hou W , Ji Z, Ji H, Hicks SC. A systematic evaluation of single-cell RNA-sequencing imputation methods. Genome Biol 2020;21:218. 32854757 10.1186/s13059-020-02132-x PMC7450705

[58] Patruno L , Maspero D, Craighero F, Angaroni F, Antoniotti M, Graudenzi A. A review of computational strategies for denoising and imputation of single-cell transcriptomic data. Brief Bioinform 2021;22:bbaa222. 33003202 10.1093/bib/bbaa222

[59] van Dijk D , Sharma R, Nainys J, Yim K, Kathail P, Carr AJ, et al Recovering gene interactions from single-cell data using data diffusion. Cell 2018;174:716–29.e27. 29961576 10.1016/j.cell.2018.05.061 PMC6771278

[60] Dumais ST. Latent semantic analysis. Annu Rev Inform Sci Technol 2004;38:188–230.

[61] Pritchard JK , Stephens M, Donnelly P. Inference of population structure using multilocus genotype data. Genetics 2000;155:945–59. 10835412 10.1093/genetics/155.2.945 PMC1461096

[62] Blei DM , Ng AY, Jordan MI. Latent dirichlet allocation. J Mach Learn Res 2003;3:993–1022.

[63] Lee DD , Seung HS. Learning the parts of objects by non-negative matrix factorization. Nature 1999;401:788–91. 10548103 10.1038/44565

[64] Borg I , Groenen PJF. Modern Multidimensional Scaling: Theory and Applications. New York: Springer; 2005.

[65] Hahsler M , Piekenbrock M, Doran D. dbscan: fast density-based clustering with R. J Stat Softw 2019;91:1–30.

[66] Blondel V , Guillaume JL, Lambiotte R, Lefebvre E. Fast unfolding of communities in large networks. J Stat Mech 2008;P10008.

[67] Waltman L , van Eck NJ. A smart local moving algorithm for large-scale modularity-based community detection. Eur Phys J B 2013;86:471.

[68] Traag VA , Waltman L, van Eck NJ. From Louvain to Leiden: guaranteeing well-connected communities. Sci Rep 2019;9:5233. 30914743 10.1038/s41598-019-41695-z PMC6435756

[69] van der Maaten L , Hinton G. Visualizing data using *t*-SNE. J Mach Learn Res 2008;9:2579–605.

[70] Becht E , McInnes L, Healy J, Dutertre CA, Kwok IWH, Ng LG, et al Dimensionality reduction for visualizing single-cell data using UMAP. Nat Biotechnol 2019;37:38–44. 10.1038/nbt.4314 30531897

[71] McInnes L , Healy J, Melville J. UMAP: uniform manifold approximation and projection for dimension reduction. arXiv 2018;1802.03426.

[72] Zhang X , Lan Y, Xu J, Quan F, Zhao E, Deng C, et al CellMarker: a manually curated resource of cell markers in human and mouse. Nucleic Acids Res 2019;47:D721–8. 30289549 10.1093/nar/gky900 PMC6323899

[73] Franzen O , Gan LM, Bjorkegren JLM. PanglaoDB: a web server for exploration of mouse and human single-cell RNA sequencing data. Database (Oxford) 2019;2019:baz046. 30951143 10.1093/database/baz046 PMC6450036

[74] Regev A , Teichmann SA, Lander ES, Amit I, Benoist C, Birney E, et al The Human Cell Atlas. Elife 2017;6:e27041. 29206104 10.7554/eLife.27041 PMC5762154

[75] Tabula Muris Consortium. Single-cell transcriptomics of 20 mouse organs creates a Tabula Muris. Nature 2018;562:367–72. 30283141 10.1038/s41586-018-0590-4 PMC6642641

[76] Wang Y , Sun X, Zhao H. Benchmarking automated cell type annotation tools for single-cell ATAC-seq data. Front Genet 2022;13:1063233. 36583014 10.3389/fgene.2022.1063233 PMC9792779

[77] Love MI , Huber W, Anders S. Moderated estimation of fold change and dispersion for RNA-seq data with DESeq2. Genome Biol 2014;15:550. 25516281 10.1186/s13059-014-0550-8 PMC4302049

[78] Robinson MD , McCarthy DJ, Smyth GK. edgeR: a Bioconductor package for differential expression analysis of digital gene expression data. Bioinformatics 2010;26:139–40. 19910308 10.1093/bioinformatics/btp616 PMC2796818

[79] Ritchie ME , Phipson B, Wu D, Hu Y, Law CW, Shi W, et al Limma powers differential expression analyses for RNA-sequencing and microarray studies. Nucleic Acids Res 2015;43:e47. 25605792 10.1093/nar/gkv007 PMC4402510

[80] Li Y , Ge X, Peng F, Li W, Li JJ. Exaggerated false positives by popular differential expression methods when analyzing human population samples. Genome Biol 2022;23:79. 35292087 10.1186/s13059-022-02648-4 PMC8922736

[81] D’Haeseleer P. What are DNA sequence motifs? Nat Biotechnol 2006;24:423–5. 16601727 10.1038/nbt0406-423

[82] Mei S , Qin Q, Wu Q, Sun H, Zheng R, Zang C, et al Cistrome Data Browser: a data portal for ChIP-Seq and chromatin accessibility data in human and mouse. Nucleic Acids Res 2017;45:D658–62. 27789702 10.1093/nar/gkw983 PMC5210658

[83] Vierstra J , Stamatoyannopoulos JA. Genomic footprinting. Nat Methods 2016;13:213–21. 26914205 10.1038/nmeth.3768

[84] Heinz S , Benner C, Spann N, Bertolino E, Lin YC, Laslo P, et al Simple combinations of lineage-determining transcription factors prime *cis*-regulatory elements required for macrophage and B cell identities. Mol Cell 2010;38:576–89. 20513432 10.1016/j.molcel.2010.05.004 PMC2898526

[85] Machanick P , Bailey TL. MEME-ChIP: motif analysis of large DNA datasets. Bioinformatics 2011;27:1696–7. 21486936 10.1093/bioinformatics/btr189 PMC3106185

[86] Castro-Mondragon JA , Riudavets-Puig R, Rauluseviciute I, Lemma RB, Turchi L, Blanc-Mathieu R, et al JASPAR 2022: the 9th release of the open-access database of transcription factor binding profiles. Nucleic Acids Res 2022;50:D165–73. 34850907 10.1093/nar/gkab1113 PMC8728201

[87] Layer RM , Pedersen BS, DiSera T, Marth GT, Gertz J, Quinlan AR. GIGGLE: a search engine for large-scale integrated genome analysis. Nat Methods 2018;15:123–6. 29309061 10.1038/nmeth.4556 PMC5872823

[88] Bentsen M , Goymann P, Schultheis H, Klee K, Petrova A, Wiegandt R, et al ATAC-seq footprinting unravels kinetics of transcription factor binding during zygotic genome activation. Nat Commun 2020;11:4267. 32848148 10.1038/s41467-020-18035-1 PMC7449963

[89] Li Z , Schulz MH, Look T, Begemann M, Zenke M, Costa IG. Identification of transcription factor binding sites using ATAC-seq. Genome Biol 2019;20:45. 30808370 10.1186/s13059-019-1642-2 PMC6391789

[90] Trapnell C , Cacchiarelli D, Grimsby J, Pokharel P, Li S, Morse M, et al The dynamics and regulators of cell fate decisions are revealed by pseudotemporal ordering of single cells. Nat Biotechnol 2014;32:381–6. 24658644 10.1038/nbt.2859 PMC4122333

[91] Street K , Risso D, Fletcher RB, Das D, Ngai J, Yosef N, et al Slingshot: cell lineage and pseudotime inference for single-cell transcriptomics. BMC Genomics 2018;19:477. 29914354 10.1186/s12864-018-4772-0 PMC6007078

[92] Chen H , Albergante L, Hsu JY, Lareau CA, Lo Bosco G, Guan J, et al Single-cell trajectories reconstruction, exploration and mapping of omics data with STREAM. Nat Commun 2019;10:1903. 31015418 10.1038/s41467-019-09670-4 PMC6478907

[93] Welch JD , Kozareva V, Ferreira A, Vanderburg C, Martin C, Macosko EZ. Single-cell multi-omic integration compares and contrasts features of brain cell identity. Cell 2019;177:1873–87.e17. 31178122 10.1016/j.cell.2019.05.006 PMC6716797

[94] Duren Z , Chen X, Zamanighomi M, Zeng W, Satpathy AT, Chang HY, et al Integrative analysis of single-cell genomics data by coupled nonnegative matrix factorizations. Proc Natl Acad Sci U S A 2018;115:7723–8. 29987051 10.1073/pnas.1805681115 PMC6065048

[95] Dou J , Liang S, Mohanty V, Miao Q, Huang Y, Liang Q, et al Bi-order multimodal integration of single-cell data. Genome Biol 2022;23:112. 35534898 10.1186/s13059-022-02679-x PMC9082907

[96] Demetci P , Santorella R, Sandstede B, Noble WS, Singh R. SCOT: single-cell multi-omics alignment with optimal transport. J Comput Biol 2022;29:3–18. 35050714 10.1089/cmb.2021.0446 PMC8812493

[97] Gong B , Zhou Y, Purdom E. Cobolt: integrative analysis of multimodal single-cell sequencing data. Genome Biol 2021;22:351. 34963480 10.1186/s13059-021-02556-z PMC8715620

[98] Li G , Fu S, Wang S, Zhu C, Duan B, Tang C, et al A deep generative model for multi-view profiling of single-cell RNA-seq and ATAC-seq data. Genome Biol 2022;23:20. 35022082 10.1186/s13059-021-02595-6 PMC8756637

[99] Gayoso A , Lopez R, Xing G, Boyeau P, Valiollah Pour Amiri V, Hong J, et al A Python library for probabilistic analysis of single-cell omics data. Nat Biotechnol 2022;40:163–6. 35132262 10.1038/s41587-021-01206-w

[100] Liu Q , Chen S, Jiang R, Wong WH. Simultaneous deep generative modeling and clustering of single cell genomic data. Nat Mach Intell 2021;3:536–44. 34179690 10.1038/s42256-021-00333-y PMC8223760

[101] Lin Y , Wu TY, Wan S, Yang JYH, Wong WH, Wang YXR. scJoint integrates atlas-scale single-cell RNA-seq and ATAC-seq data with transfer learning. Nat Biotechnol 2022;40:703–10. 35058621 10.1038/s41587-021-01161-6 PMC9186323

[102] Epi Multiome ATAC + Gene Expression. 10X Genomics. https://www.10xgenomics.com/products/epi-multiome (11/18/2025 last accessed).

[103] Ma S , Zhang B, LaFave LM, Earl AS, Chiang Z, Hu Y, et al Chromatin potential identified by shared single-cell profiling of RNA and chromatin. Cell 2020;183:1103–16.e20. 33098772 10.1016/j.cell.2020.09.056 PMC7669735

[104] Hao Y , Hao S, Andersen-Nissen E, Mauck WM 3rd, Zheng S, Butler A, et al Integrated analysis of multimodal single-cell data. Cell 2021;184:3573–87.e29. 34062119 10.1016/j.cell.2021.04.048 PMC8238499

[105] Lynch AW , Theodoris CV, Long HW, Brown M, Liu XS, Meyer CA. MIRA: joint regulatory modeling of multimodal expression and chromatin accessibility in single cells. Nat Methods 2022;19:1097–108. 36068320 10.1038/s41592-022-01595-z PMC9517733

[106] Przytycki PF , Pollard KS. CellWalker integrates single-cell and bulk data to resolve regulatory elements across cell types in complex tissues. Genome Biol 2021;22:61. 33583425 10.1186/s13059-021-02279-1 PMC7883575

[107] Dong X, Tang K, Xu Y, Wei H, Han T, Wang C. Single-cell gene regulation network inference by large-scale data integration. Nucleic Acids Res 2022;50:e126. 10.1093/nar/gkac819 PMC9756951 36155797

[108] Tarhan L , Bistline J, Chang J, Galloway B, Hanna E, Weitz E. Single Cell Portal: an interactive home for single-cell genomics data. bioRxiv 2023;548886.

[109] Megill C , Martin B, Weaver C, Bell S, Prins L, Badajoz S, et al cellxgene: a performant, scalable exploration platform for high dimensional sparse matrices. bioRxiv 2021;438318.

[110] Qian FC , Zhou LW, Zhu YB, Li YY, Yu ZM, Feng CC, et al scATAC-Ref: a reference of scATAC-seq with known cell labels in multiple species. Nucleic Acids Res 2024;52:D285–92. 37897340 10.1093/nar/gkad924 PMC10767920

[111] Zhao Y , Yu ZM, Cui T, Li LD, Li YY, Qian FC, et al scBlood: a comprehensive single-cell accessible chromatin database of blood cells. Comput Struct Biotechnol J 2024;23:2746–53. 39050785 10.1016/j.csbj.2024.06.015 PMC11266868

[112] Chen Z , Zhang J, Liu J, Zhang Z, Zhu J, Lee D, et al SCAN-ATAC-Sim: a scalable and efficient method for simulating single-cell ATAC-seq data from bulk-tissue experiments. Bioinformatics 2021;37:1756–8. 33471102 10.1093/bioinformatics/btaa1039 PMC8289380

[113] Navidi Z , Zhang L, Wang B. simATAC: a single-cell ATAC-seq simulation framework. Genome Biol 2021;22:74. 33663563 10.1186/s13059-021-02270-w PMC7934446

[114] Cakir B , Prete M, Huang N, van Dongen S, Pir P, Kiselev VY. Comparison of visualization tools for single-cell RNAseq data. NAR Genom Bioinform 2020;2:lqaa052. 32766548 10.1093/nargab/lqaa052 PMC7391988

[115] Gardeux V , David FPA, Shajkofci A, Schwalie PC, Deplancke B. ASAP: a web-based platform for the analysis and interactive visualization of single-cell RNA-seq data. Bioinformatics 2017;33:3123–5. 28541377 10.1093/bioinformatics/btx337 PMC5870842

[116] Speir ML , Bhaduri A, Markov NS, Moreno P, Nowakowski TJ, Papatheodorou I, et al UCSC Cell Browser: visualize your single-cell data. Bioinformatics 2021;37:4578–80. 34244710 10.1093/bioinformatics/btab503 PMC8652023

[117] Wang Z , Zhang Y, Yu Y, Zhang J, Liu Y, Zou Q. A unified deep learning framework for single-cell ATAC-seq analysis based on ProdDep transformer encoder. Int J Mol Sci 2023;24:4784. 36902216 10.3390/ijms24054784 PMC10003007

[118] Yuan Q , Duren Z. Inferring gene regulatory networks from single-cell multiome data using atlas-scale external data. Nat Biotechnol 2025;43:247–57. 38609714 10.1038/s41587-024-02182-7 PMC11825371

[119] Yang Z , Fan X, Lan M, Li X, You Y, Tian L, et al Multiomic foundation model predicts epigenetic regulation by zero-shot. bioRxiv 2024;629561.

[120] Wu J , Wan C, Ji Z, Zhou Y, Hou W. EpiFoundation: a foundation model for single-cell ATAC-seq via peak-to-gene alignment. bioRxiv 2025;636688.

[121] Cui H , Wang C, Maan H, Pang K, Luo F, Duan N, et al scGPT: toward building a foundation model for single-cell multi-omics using generative AI. Nat Methods 2024;21:1470–80. 38409223 10.1038/s41592-024-02201-0

[122] Jiao Y , Liu Y, Zhang Y, Guo X, Wu Y, Jiang C, et al ChromFound: towards a universal foundation model for single-cell chromatin accessibility data. arXiv 2025;2505.12638.

[123] Han X , Wu H, Wang X, Liu D, Fu Y, Yang L, et al Modeling the vertebrate regulatory sequence landscape by UUATAC-seq and deep learning. Cell 2025;188:5343–62.e29. 40633538 10.1016/j.cell.2025.06.020

[124] Farzad N , EnninfulA, BaoS, ZhangD, DengY, FanR. Spatially resolved epigenome sequencing via Tn5 transposition and deterministic DNA barcoding in tissue. Nat Protoc 2024;19:3389–425.38943021 10.1038/s41596-024-01013-y

[125] Llorens-Bobadilla E , Zamboni M, Marklund M, Bhalla N, Chen X, Hartman J, et al Solid-phase capture and profiling of open chromatin by spatial ATAC. Nat Biotechnol 2023;41:1085–8. 36604544 10.1038/s41587-022-01603-9 PMC10421738

[126] Thornton CA , Mulqueen RM, Torkenczy KA, Nishida A, Lowenstein EG, Fields AJ, et al Spatially mapped single-cell chromatin accessibility. Nat Commun 2021;12:1274. 33627658 10.1038/s41467-021-21515-7 PMC7904839

[127] Fang S , Chen B, Zhang Y, Sun H, Liu L, Liu S, et al Computational approaches and challenges in spatial transcriptomics. Genomics Proteomics Bioinformatics 2023;21:24–47. 36252814 10.1016/j.gpb.2022.10.001 PMC10372921

[128] Li H , BaoS, FarzadN, QinX, FungAA, ZhangD, et al Spatially resolved genome-wide joint profiling of epigenome and transcriptome with spatial-ATAC-RNA-seq and spatial-CUT&Tag-RNA-seq. Nat Protoc 2025;20:2383–417.40119005 10.1038/s41596-025-01145-9

[129] Chen X , Li K, Wu X, Li Z, Jiang Q, Cui X, et al Descart: a method for detecting spatial chromatin accessibility patterns with inter-cellular correlations. Genome Biol 2024;25:322. 39736655 10.1186/s13059-024-03458-6 PMC11686967

[130] Yang P , Jin K, Yao Y, Jin L, Shao X, Li C, et al Spatial integration of multi-omics single-cell data with SIMO. Nat Commun 2025;16:1265. 39893194 10.1038/s41467-025-56523-4 PMC11787318

[131] Dhapola P , Rodhe J, Olofzon R, Bonald T, Erlandsson E, Soneji S, et al Scarf enables a highly memory-efficient analysis of large-scale single-cell genomics data. Nat Commun 2022;13:4616. 35941103 10.1038/s41467-022-32097-3 PMC9360040

[132] Wang X , Lian Q, Dong H, Xu S, Su Y, Wu X. Benchmarking algorithms for geneset scoring of single-cell ATAC-seq data. Genomics Proteomics Bioinformatics 2024;22:qzae014. 39049508 10.1093/gpbjnl/qzae014 PMC11423854

[133] scRNA-seq and scATAC-seq Data Analysis DREAM Challenge. Synapse. https://www.synapse.org/Synapse:syn26720920/wiki/ (11/18/2025 last accessed).

[134] Burkhardt D , Luecken M, Benz A, Holderrieth P, Bloom J, Lance C, et al Open Problems—Multimodal Single-Cell Integration. Kaggle. https://kaggle.com/competitions/open-problems-multimodal (11/18/2025 last accessed).

[135] Tarbell ED , Liu T. HMMRATAC: a Hidden Markov ModeleR for ATAC-seq. Nucleic Acids Res 2019;47:e91. 31199868 10.1093/nar/gkz533 PMC6895260

