# Supplementary material for: Computational Analyses and Challenges of Single-cell ATAC-seq
Source: Genomics Proteomics Bioinformatics. 2025 Nov 21;23(6):qzaf115. doi: 10.1093/gpbjnl/qzaf115 (PMC12753137; doi:10.1093/gpbjnl/qzaf115)
Supplement: qzaf115_Supplementary_Data [file qzaf115_supplementary_data.zip › Supplementary material captions.docx]

**Table S1 A curated summary of representative scATAC-seq analysis packages**

**Table S2 A curated summary of scATAC-seq databases**
